# Protocol of a randomized controlled trial investigating Deep Brain Stimulation for MOtor symptoms in patients with Parkinson’s disease DEmentia (DBS-MODE)

**DOI:** 10.1186/s12883-023-03142-5

**Published:** 2023-04-21

**Authors:** V. Sisodia, B. E. K. S. Swinnen, J. M. Dijk, E. Verwijk, G. van Rooijen, A. W. Lemstra, P. R. Schuurman, R. M. A. de Bie

**Affiliations:** 1 Amsterdam UMC location University of Amsterdam, Neurology, Meibergdreef 9, Amsterdam, Netherlands; 2 Amsterdam Neuroscience, Neurodegeneration, Amsterdam, Netherlands; 3 Amsterdam UMC location University of Amsterdam, Medical Psychology, Amsterdam, Netherlands; 4 Amsterdam UMC location University of Amsterdam, Psychiatry, Amsterdam, Netherlands; 5 Amsterdam UMC location Vrije Universiteit Amsterdam, Neurology, De Boelelaan, 1117 Amsterdam, Netherlands; 6 Amsterdam UMC location University of Amsterdam, Neurosurgery, Amsterdam, Netherlands

**Keywords:** Parkinson’s disease, Dementia, Deep brain stimulation, Randomized controlled trial

## Abstract

**Background:**

Deep brain stimulation (DBS) of the subthalamic nucleus (STN) is an established treatment for disabling motor symptoms of Parkinson’s disease (PD) that persist despite optimal pharmacological treatment. Currently, DBS is not performed if there is concomitant significant cognitive impairment based on concerns of cognitive deterioration, higher complication rate and less functional improvement. However, this has not been investigated so far.

**Methods:**

A single center, prospective, randomized, open-label, blinded end-point (PROBE design) pilot clinical trial is being performed. Patients are eligible for the trial if they have PD dementia (PDD), are able to provide informed consent, and experience disabling motor response fluctuations, bradykinesia, dyskinesia, or painful dystonia, despite optimal pharmacological treatment. In total 44 patients will be randomized to either STN-DBS accompanied by best medical treatment (DBS group) or to best medical treatment alone (BMT group). The primary outcome measure is the change from baseline to 30 weeks on the Movement Disorder Society—Unified Parkinson’s Disease Rating Scale part III score in a standardized off-drug phase. The main secondary outcome measures consist of scales assessing cognitive aspects of daily living, neuropsychiatric symptoms and impulsive compulsive disorders. Additional secondary outcome measures include motor signs during on-drug phase, dyskinesia, motor fluctuations, cognitive performance, (severe) adverse events, treatment satisfaction, and caregiver burden. Patients will be followed during 52 weeks after randomization.

**Discussion:**

The Deep Brain Stimulation for MOtor symptoms in patients with Parkinson’s disease DEmentia (DBS-MODE) trial directly compares the effectiveness and safety of DBS with BMT in patients with PDD.

**Trial registration:**

The DBS-MODE trial has been registered in the International Clinical Trial Registry Platform (NL9361) on the 24^th^ of March 2021 (https://trialsearch.who.int/Trial2.aspx?TrialID=NL9361).

**Supplementary Information:**

The online version contains supplementary material available at 10.1186/s12883-023-03142-5.

## Background

Parkinson’s disease (PD) is a neurodegenerative disorder that affects the motor, autonomic, cognitive, affective, and sensory systems [1]. Deep brain stimulation (DBS) of the subthalamic nucleus (STN) is an established treatment for disabling motor response fluctuations and dyskinesia that persist despite optimal pharmacological treatment [2]. After DBS surgery, patients with advanced PD generally have significant improvement of motor symptoms, functioning in daily living, and health-related quality of life (QoL). Cognitive impairment is an important and disabling feature of PD. Within ten years after diagnosis up to 70% of patients with PD will have developed PD dementia (PDD); meaning there is an obvious deficit in at least two cognitive domains impairing functioning in daily life [3, 4].

Currently, PDD is considered a relative contra-indication for treatment of motor symptoms with DBS [5]. There is a concern that DBS might worsen cognitive impairments and that the former generally routine procedure of the patient being awake during DBS surgery might be too burdensome for patients with dementia. Yet, the rationale to withhold these patients from DBS is weak and current literature even suggests otherwise. First, PD patients with mild cognitive impairment (MCI) treated with DBS do not experience a significant additional cognitive decline compared to patients who have not undergone DBS [6, 7]. The impact of DBS on cognition and development of delirium and psychosis in patients with PDD however is unclear. The current evidence consists of a case series, in which this did not seem to be an obvious issue [8]. In patients with PD who underwent DBS surgery of the nucleus basalis of Meynert aimed at improving cognitive symptoms, the risk of developing delirium and psychosis did not appear to be increased [9–11]. Second, the burden of DBS surgery has been significantly reduced now that DBS surgery of the STN under general anesthesia is becoming standard-of-care and seems equally effective for motor symptom control [12–14]. Lastly, the presence of dementia is not an absolute exclusion criterion for other (invasive) procedures, such as surgery after hip fracture and dialysis for end stage renal disease [15, 16].

The improvement in motor function with DBS in PDD patients is possibly similar to PD patients without dementia [8, 17]. However, this has never been formally tested in this population and hence this is the primary objective of this pilot trial. Our main secondary objective is to investigate possible changes in cognitive functioning and psychiatric symptoms following DBS compared with best oral medical treatment (BMT). The findings of this pilot trial will help determining the merits of conducting a future large-scale clinical trial to assess the clinical usefulness of DBS in PDD.

## Methods

### Study design

**Fig. 1:**
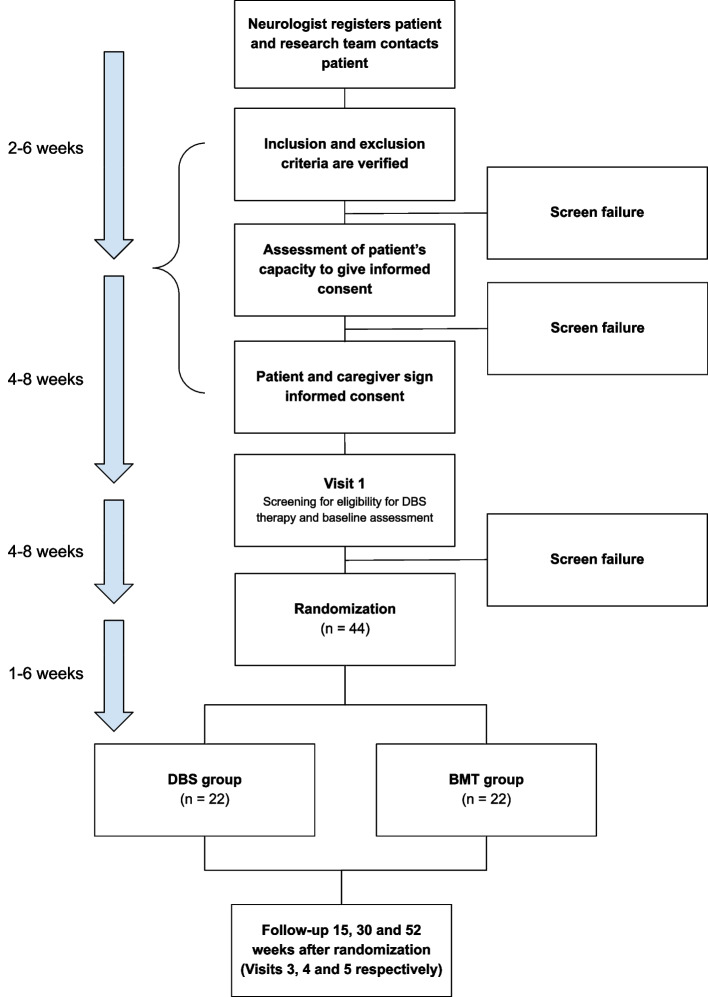
Flowchart of the DBS-MODE trial. Deep brain stimulation (DBS) group: treatment with DBS of the subthalamic nucleus and best medical treatment (BMT). BMT group: treatment with BMT

The study is a single center, prospective, randomized, open-label, blinded end-point (PROBE design) pilot clinical trial. The effectiveness and safety of STN DBS surgery with current standard practice of medical treatment will be assessed (Fig. 1).

### Patients

Patients will be recruited from Amsterdam UMC, The Netherlands. Patients with PD and disabling motor symptoms who are referred to Amsterdam UMC for DBS screening and who have dementia will be asked to participate in the trial. In order to participate in this study, a subject must meet all of the following inclusion criteria: a) age 18 years and older; b) diagnosis of PD according to the clinical diagnostic criteria of the Movement Disorder Society (MDS) [18]; c) despite optimal pharmacological treatment, at least one of the following symptoms, that are severe enough to impair functioning in daily life independent of dementia: motor response fluctuations, dyskinesia, painful dystonia, or levodopa-responsive bradykinesia; d) diagnosis of probable or possible PDD according to the MDS clinical diagnostic criteria [3] (see supplementary Table 1); e) a life expectancy of at least two years; f) subject has decision capacity to give informed consent; g) subject provides written informed consent; h) regular contact with a caregiver, who has on average at least twice a week contact with the subject and also provides written informed consent for their own participation. Exclusion criteria are: a) any neurodegenerative disorder other than PD; b) previous neurosurgery for PD (*e.g.*, DBS, pallidotomy, thalamotomy); c) contraindications for DBS surgery, such as a physical disorder making surgery hazardous; d) Hoehn and Yahr stage 4 or 5 at the best moment during the day [19]; e) co‐existence of another abnormality or disorder, that causes cognitive impairment that may improve with specific treatment or that besides PDD is judged to contribute significantly to the cognitive impairment by the treating physician; f) current major depressive episode according to the fifth edition of the Diagnostic and Statistical Manual of Mental Disorders (DSM-5) [20]; g) current psychosis; h) other severely disabling condition; i) immobility during the greater part of the day not related to off-drug phase (*e.g.*, due to apathy); j) pregnancy, breastfeeding, and women of childbearing age not using a reliable method of contraception. Of importance, previous treatment with intrajejunal levodopa infusion or subcutaneous apomorphine infusion is not a reason for exclusion.

### Study procedures

Patients with PD and dementia who have been referred to Amsterdam UMC for DBS, will be informed about the study and will be asked to participate in the study. The research team will decide if patients are considered eligible based on the inclusion and exclusion criteria. Many patients with dementia are able to give informed consent [21, 22]. However, the assessment of the capacity to give informed consent will be done in advance at the discretion of an experienced neurologist from the study team. If a potentially eligible patient is considered not to be able to provide consent because he or she is mentally incompetent, the patient will not be included in the trial. In order to be eligible to participate in this study, it is necessary that the patient has an involved caregiver who will be present during the follow-up visits. Because the caregiver is partaking in the study, the caregiver also needs to sign an informed consent for his or her own participation. After written informed consent has been obtained, patients will be assessed in-hospital whether improvement in motor symptoms is considered likely by means of standardized on- and off-drug assessments. This will be done during Visit 1 along with the assessment of other study outcomes. The results of Visit 1 will be discussed during a multidisciplinary meeting, in which the DBS indication for the treatment of motor symptoms is discussed. This is similar to standard care. If patients are considered eligible, they will be randomized to treatment with STN-DBS and BMT (DBS group) or to BMT without the operation (BMT group).

### Randomization

In this trial, 44 patients will be randomized to the DBS group or BMT group in a 1:1 ratio. The randomization of the patients will be web-based and performed on Castor by using randomly permuted blocks with block sizes of 2 and 4 [23]. There is no stratification of randomization. Blinded assessment of the primary outcome (*i.e.*, Movement Disorder Society-Unified Parkinson’s Disease Rating Scale (MDS–UPDRS) part III during off-drug phase) will be performed by a blinded study team member. Because in bald patients and patients with short hair stigmata due to the DBS treatment may be visible, all patients will wear a head cover during the motor examinations at baseline and 30 weeks follow-up.

### Intervention

#### Deep brain stimulation surgery

Implantation of the DBS electrodes will be performed under general anesthesia with a stereotactic frame-based procedure. Target coordinates are obtained after fusion of an intraoperative cone-beam CT with a pre-operative 3-T MRI with the target planning. One-track microelectrode recordings are performed to confirm the dorsal and ventral border of the STN. After electrode implantation, the electrodes are connected via extension cables to a pulse generator that is implanted on the wall of the chest in the same surgical session. Confirmation of correct electrode placement is performed with a direct intraoperative CT-scan.

The pulse generator is connected to the electrodes. On average, patients are hospitalized for four days. Patients will regularly visit the outpatient clinic to adjust stimulation parameters and PD medication as part of the regular clinical routine. If necessary, caregivers may adjust the stimulation parameters with a DBS remote control after contacting the DBS team by telephone to minimize the burden of the patient. The protocol does not impose the use of DBS equipment from a particular manufacturer.

#### Best oral medical treatment

All patients will receive BMT, consisting of PD treatment according to current guidelines and can be adjusted accordingly. This includes all modes of medications (*e.g.*, levodopa, dopamine agonists, monoamine oxidase type B inhibitors, catechol-O-methyltransferase inhibitors, and amantadine), except advanced device-assisted treatments (*e.g.*, continuous intrajejunal levodopa infusion and subcutaneous apomorphine infusion) unless already initiated before inclusion. The referring treating neurologist will manage the medical treatment. If necessary, neurologists in Amsterdam UMC will advise the treating neurologist on how to optimize the medical treatment.

### Objectives

As previously mentioned, all objectives in this study serve to guide the conduct of a potential future larger-scale trial evaluating the clinical usefulness of DBS in PDD. The primary objective is to answer the following question: do patients with PDD who are suffering from disabling motor response fluctuations or dyskinesia experience a clinically relevant improvement in motor impairment during off-drug phase after treatment with DBS of the STN accompanied by BMT compared to BMT alone?

Our main secondary objective is to investigate the difference in cognitive impairment and psychiatric symptoms between the DBS group and the BMT group. Other secondary objectives include on-drug phase motor symptoms, functional health status, incidence of falls, use of PD medication, (serious) adverse events ((S)AEs), treatment satisfaction, caregiver burden and medical care use compared between the DBS group and BMT group.

### Outcome measures and assessment scales

#### Primary outcome measures

The primary outcome is the change from baseline to 30 weeks after randomization in MDS-UPDRS part III off-drug phase [24] compared between the DBS group and the BMT group.

#### Secondary outcome measures

The main secondary outcomes are: a) cognitive evaluation (Amsterdam iADL questionnaire – short version (A-IADL-Q-SV) [25]); and b) psychiatric assessment (Neuropsychiatric Inventory Questionnaire (NPI-Q) [26] and Questionnaire for Impulsive Disorders in Parkinson’s Disease (QUIP) [27]). For the main secondary outcome a composite outcome measure will be used. Fulfilling one or more of the criteria for this composite outcome measure is compatible with cognitive and/or psychiatric decline. Patients will fulfill the criteria for this composite outcome measure if at least one of the following applies: a) the post-operative A-IADL-Q-SV score is ≥ 2.50 points lower than the pre-operative score; b) regarding the NPI-Q: the post-operative severity score on one of the 12 items is ≥ 2 points higher than the pre-operative score or the post-operative total NPI-Q severity score is ≥ 4 points higher than the pre-operative score; or c) on the post-operative QUIP the patient fulfills the criteria for either an impulse control disorder (ICD), other compulsive behavior or compulsive use of medication when these criteria were not fulfilled pre-operatively. Additional secondary outcomes are: a) motor symptoms (*i.e.*, MDS-UPDRS part III in on-drug phase, MDS-UPDRS part II in both off-drug and on-drug phase, Clinical Dyskinesia Rating Scale (CDRS) [28], and motor symptom diary); b) cognitive evaluation (Montreal Cognitive Assessment (MoCA) [29]); c) functional health (Academic Medical Center Linear Disability Score (ALDS) [30], Modified Rankin Scale (MRS) [31], Hoehn and Yahr stage, and Clinical Global Impression of Change (CGI-C)); d) incidence of falls in the preceding three months; e) PD and psychiatric medication; f) (serious) adverse events ((S)AE, including but not limited to surgical complications, delirium, hospitalization, and admission in nursing home; g) caregiver burden (Zarit Burden Interview [32]); h) recruitment and retention rate; and i) medical care use, which will be estimated by the amount of additional medical care contacts (*i.e.*, outpatient clinic, inpatient clinic and telephone consultation). Other study parameters that will only be assessed in the DBS group are the occurrence of delirium in the postoperative phase (based on DSM-5 criteria) and treatment satisfaction. The manufacturers and types of DBS systems that are used will also be reported. For an overview of all assessments, see supplementary Table 2.

### Assessment visits

**Fig. 2:**
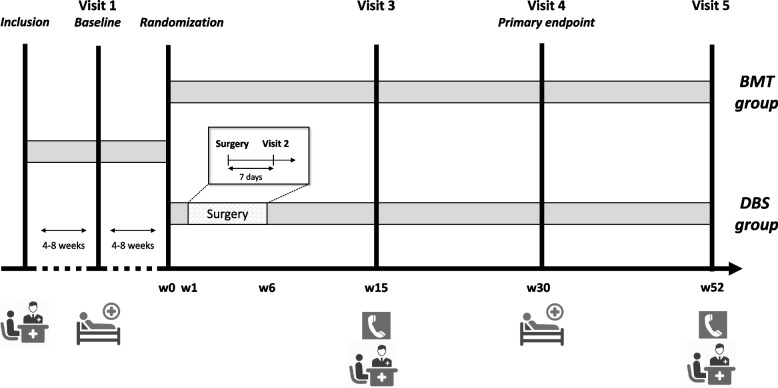
Timeline of study visits. Deep brain stimulation (DBS) group: treatment with DBS of the subthalamic nucleus and best medical treatment (BMT). BMT group: treatment with BMT

There are six prespecified assessment visits (Fig. 2); an inclusion visit (outpatient clinic), a baseline assessment (Visit 1—hospitalization), an assessment one week after surgery (Visit 2 – outpatient clinic), an assessment (of safety) 15 weeks after randomization (Visit 3 – telephone or outpatient clinic), an assessment of the primary and secondary endpoints 30 weeks after randomization (Visit 4—hospitalization), and an assessment of functional health status 52 weeks after randomization (Visit 5 – telephone or outpatient clinic). See supplementary Table 2 for the assessments performed at the different visits.

### Statistics

#### Sample size calculation

The sample size calculation is based on the primary outcome measure. Given the (cognitive) vulnerability of the study population and as it is unknown whether the improvement in motor symptoms can outweigh the possible deterioration in cognition, a relatively large between-group difference in favor of DBS is needed to justify the use of DBS. An improvement of 15 points on the MDS-UPDRS part III in the off-drug state is assumed to be minimally clinically important. Therefore, a sample size of 17 patients in each group (34 patients in total) will have 80% power to detect a difference in mean change scores of 15 points on the MDS-UPDRS part III, assuming that the common standard deviation is 15, using a two-group t-test with a 5% two-sided significance level. Anticipating a 20% attrition rate, 22 patients per treatment arm will be included (44 patients in total).

For the main secondary objectives, the sample size has enough power only to detect large between-group differences in proportions of cognitive and psychiatric problems. Assuming a proportion of 10% of patients in the BMT group fulfilling the criteria for the composite outcome measure, a Fisher’s exact test with a 5% two-sided significance, will have 80% power to detect a difference of 49% between the DBS and BMT group when the sample size in each group is 17.

#### Statistical analysis

We will prepare a detailed statistical analysis plan before the database is finalized and locked. The statistical analyses will be based on the intention-to-treat principle. Baseline characteristics will be summarized using descriptive statistics. In all analyses statistical uncertainties will be expressed in 95% confidence intervals. A two-sided p-value < 0.05 will be considered statistically significant. The primary outcome - the MDS-UPDRS part III score in off-drug phase at week 30 - will be analyzed using the two-group t-test, assuming normal distribution. Additionally, the treatment effects on the MDS-UPDRS follow-up scores will be analyzed, using multivariable linear regression, including the baseline MDS-UPDRS scores into the model.

For the main secondary outcome, a composite dichotomous outcome measure will be used. The difference in the proportion for the dichotomous composite outcome measure between the DBS and BMT group will be analyzed using the chi-squared test or Fisher’s exact test, when appropriate. For the other secondary outcomes, we will use the appropriate parametric and nonparametric statistics to compare the DBS group and the BMT group for normally and not-normally distributed data, respectively.

#### Data safety analysis

As additional risks are associated with this trial, a data safety monitoring board (DSMB) will be established. This committee is independent and has no conflict of interest with the sponsor of the study. During the trial DSMB meetings will be held two months after the fifth, tenth and fifteenth patient in the DBS group have undergone surgery. During these meetings relevant baseline data, study performance data, secondary outcome data and safety data will be analysed and reviewed. Primary outcome data will not be reviewed. Since the interim analysis concerns an analysis for safety in a small sample, no pre-specified formal statistical stopping rule for safety is formulated.

## Discussion

A pilot trial investigating the motor efficacy as well as cognitive and psychiatric outcomes of DBS for patients with PDD is necessary and timely. Currently, patients with PD and disabling motor symptoms who are also suffering from dementia are not considered for DBS. This concerns a relatively large group of patients and there are no appropriate scientific data underpinning this clinical practice. If motor function improves in PDD patients, these patients may also experience significant improvement in their QoL, there might be less burden of care and patients might even be able to live longer at home instead of in a nursing home.

Some aspects related to the study design warrant discussion. First is the study design. In order to investigate the clinical usefulness of STN-DBS in PDD, a large clinical trial is needed. However, it is unknown what the inclusion rate will be and whether these patients are able to complete such a trial. It would also be unethical to perform such a large trial before the motor benefit of DBS in PDD has been confirmed. As PDD patients constitute a vulnerable population, we aim to first assess the motor efficacy of DBS, which is the scope of this trial. All outcomes in this single-center pilot trial will be helpful for the realization of future trials of DBS in this patient population. Second, neither patients nor the referring treating neurologist can be blinded to treatment allocation. A sham surgery would be unethical. DBS surgery is an invasive procedure and patients randomized to the sham surgery would be exposed to operative risks with no possibility of benefit. Similarly, a study design where DBS is implanted in all patients, but not activated until after the trial for patients in the control group, is for the same reason not possible. Furthermore, hippocampal atrophy with brain MRI, which would suggest Alzheimer’s disease as a cause for the dementia, was not an exclusion criterion for the trial. For all patients, the PDD diagnosis according to MDS clinical diagnostic criteria was established by means of a full neuropsychological examination. Lastly, for the main secondary outcome the composite outcome measure will be used. This is not a validated outcome measure. However, the composite outcome improves the ability to detect differences in cognitive function and psychiatric symptoms between the BMT group and DBS group.

In conclusion, we have developed a protocol for a prospective, randomized, open-label pilot trial comparing motor efficacy and safety of STN DBS and BMT in patients with PDD. With the proposed study, we aim to investigate whether STN-DBS is an effective treatment for motor symptoms in PDD patients, like it is the case in non-demented PD patients. Whether this motor improvement outweighs surgical, cognitive and psychiatric safety is an exploratory outcome and might need to be assessed more extensively in a larger trial.

## Supplementary Information

**Additional file 1.**

## Data Availability

Depending on the type of data and associated privacy regulations, data from the DBS-MODE project will be made publicly available or will become available via the corresponding author, upon reasonable request.

## Abbreviations

A-IADL-Q-SV: Amsterdam iADL questionnaire – short version
ALDS: Academic Medical Center Linear Disability Score
BMT: Best oral medical treatment
CDRS: Clinical Dyskinesia Rating Scale
CGI-C: Clinical Global Impression of Change
DBS: Deep brain stimulation
DSM-5: Fifth edition of the Diagnostic and Statistical Manual of Mental Disorders
DSMB: Data safety monitoring board
ICD: Impulse control disorder
MCI: Mild cognitive impairment
MDS: Movement Disorder Society
MoCA: Montreal Cognitive Assessment
MRS: Modified Rankin Scale
NPI-Q: Neuropsychiatric Inventory Questionnaire
PD: Parkinson’s disease
PDD: Parkinson’s disease dementia
PROBE: Prospective, randomized, open-label, blinded end-point
QoL: Quality of life
QUIP: Questionnaire for Impulsive Disorders in Parkinson’s Disease
(S)AEs: (Serious) adverse events
STN: Subthalamic nucleus
UPDRS: Unified Parkinson’s Disease Rating Scale
