# Supplementary material for: Protocol of a randomized controlled trial investigating Deep Brain Stimulation for MOtor symptoms in patients with Parkinson’s disease DEmentia (DBS-MODE)
Source: BMC Neurol. 2023 Apr 21;23:160. doi: 10.1186/s12883-023-03142-5 (PMC10120102; doi:10.1186/s12883-023-03142-5)
Supplement: Supplementary file 1 — Additional file 1. [file 12883_2023_3142_MOESM1_ESM.docx]

**Supplement to: Protocol of a randomized controlled trial investigating Deep Brain Stimulation for MOtor symptoms in patients with Parkinson’s disease DEmentia (DBS-MODE)**

**V. Sisodia^1,2^, B.E.K.S. Swinnen^1,2^, J.M. Dijk^1,2^, E. Verwijk^3^, G. van Rooijen^2,4^, A. Lemstra^2,5^, P.R. Schuurman^2,6^, R.M.A de Bie^1,2^***

*** Correspondence:** [**r.m.debie@amsterdamumc.nl**](mailto:r.m.debie@amsterdamumc.nl)

**^1^** Amsterdam UMC location University of Amsterdam, Neurology, Meibergdreef 9, Amsterdam, Netherlands

**^2^** Amsterdam Neuroscience, Neurodegeneration, Amsterdam, Netherlands

**^3^** Amsterdam UMC location University of Amsterdam, Medical Psychology, Meibergdreef 9, Amsterdam, Netherlands

^4^ Amsterdam UMC location University of Amsterdam, Psychiatry, Meibergdreef 9, Amsterdam, Netherlands

^5^ Amsterdam UMC location Vrije Universiteit Amsterdam, Neurology, De Boelelaan 1117,

Amsterdam, Netherlands

^6^ Amsterdam UMC location University of Amsterdam, Neurosurgery, Meibergdreef 9, Amsterdam, Netherlands

| **Table 1: MDS diagnostic criteria of probable and possible PDD** | |
| --- | --- |
| **I. Core features** |  |
| 1. Diagnosis of Parkinson’s disease according to Queen Square Brain Bank Criteria  2. A dementia syndrome with insidious onset and slow progression, developing within the context of established Parkinson’s disease and diagnosed by history, clinical and mental examination, defined as:   - Impairment in more than one cognitive domain - Representing a decline from premorbid level - Deficits severe enough to impair daily life (social, occupational, or personal care), independent of the impairment ascribable to motor of autonomic symptoms |  |
| **II. Associated clinical features** |  |
| 1. Cognitive features:   - Attention: Impaired. Impairment in spontaneous and focused attention, poor performance in attentional tasks; performance may fluctuate during the day and from day to day. - Executive functions: Impaired. Impairment in tasks requiring initiation, planning, concept formation, rule finding, set shifting or set maintenance; impaired mental speed (bradyphrenia) - Visuo-spatial functions: Impaired. Impairment in tasks requiring visual-spatial orientation, perception, or construction - Memory: Impaired. Impairment in free recall of recent events or in tasks requiring learning new material, memory usually improves with cueing, recognition is usually better than free recall - Language: Core functions largely preserved. Word finding difficulties and impaired comprehension of complex sentences may be present   2. Behavioral features:   - Apathy: decreased spontaneity; loss of motivation, interest, and effortful behavior - Changes in personality and mood including depressive features and anxiety - Hallucinations: mostly visual, usually complex, formed visions of people, animals or objects - Delusions: usually paranoid, such as infidelity, or phantom boarder (unwelcome guests living in the home) delusions - Excessive daytime sleepiness |  |
| **III.**  **Features which do not exclude PDD, but make the diagnosis uncertain** |  |
| - Co-existence of any other abnormality which may by itself cause cognitive impairment, but judged not to be the cause of dementia, e.g. presence of relevant vascular disease in imaging - Time interval between the development of motor and cognitive symptoms not known |  |
| **IV. Features suggesting other conditions or diseases as cause of mental impairment, which, when present make it impossible to reliably diagnose PDD** |  |
| - Cognitive and behavioral symptoms appearing solely in the context of other conditions such as:   Acute confusion due to  a. Systemic diseases or abnormalities  b. Drug intoxication  Major Depression according to DSM IV   - Features compatible with “Probable Vascular dementia” criteria according to NINDS-AIREN (dementia in the context of cerebrovascular disease as indicated by focal signs in neurological exam such as hemiparesis, sensory deficits, and evidence of relevant cerebrovascular disease by brain imaging AND a relationship between the two as indicated by the presence of one or more of the following: onset of dementia within 3 months after a recognized stroke, abrupt deterioration in cognitive functions, and fluctuating, stepwise progression of cognitive deficits) |  |
| **Probable PDD** |  |
| 1. Core features: Both must be present 2. Associated clinical features:  - Typical profile of cognitive deficits including impairment in at least two of the four core cognitive domains (impaired attention which may fluctuate, impaired executive functions, impairment in visuo-spatial functions, and impaired free recall memory which usually improves with cueing) - The presence of at least one behavioral symptom (apathy, depressed or anxious mood, hallucinations, delusions, excessive daytime sleepiness) supports the diagnosis of Probable PDD, lack of behavioral symptoms, however, does not exclude the diagnosis  1. None of the group III features present 2. None of the group IV features present |  |
| **Possible PDD** |  |
| 1. Core features: Both must be present 2. Associated clinical features:  - Atypical profile of cognitive impairment in one or more domains, such as prominent or receptive-type (fluent) aphasia, or pure storage-failure type amnesia (memory does not improve with cueing or in recognition tasks) with preserved attention - Behavioral symptoms may or may not be present   OR   1. One or more of the group III features present 2. None of the group IV features present |  |
| *DSM, Diagnostic and Statistical Manual of Mental Disorders; PDD, Parkinson’s disease dementia.* |  |

| **Table 2: assessment schedule of the DBS-MODE trial** | | | | | | | |
| --- | --- | --- | --- | --- | --- | --- | --- |
| **Outcome** | **Assessment^1^** | **Assessments per visit** | | | | | **Data collection method** |
|  |  | *Visit*  *1*  *Baseline* | *Visit 2** | *Visit*  *3*  *15w* | *Visit*  *4*  *30w* | *Visit*  *5*  *52w* |  |
|  | Baseline characteristics^2^ | X |  |  |  |  | Self-reported questionnaire of patient and caregiver |
| Main outcome measure |  |  |  |  |  |  |  |
| Motor symptoms | MDS-UPDRS part III (1), motor score in off-drug phase | X |  |  | X |  | Physical examination (blinded) |
| Main secondary outcome measures |  |  |  |  |  |  |  |
| Cognition | A-IADL-Q-SV Scale (2) | X |  |  | X | X | Self-reported questionnaire of caregiver |
| Neuropsychiatric symptoms | Neuropsychiatric  Inventory Questionnaire (3) | X |  |  | X |  | Interviewed/self-reported questionnaire of caregiver |
| Impulse control disorders | Questionnaire for Impulsive-Compulsive Disorders in Parkinson’s Disease (4) | X |  |  | X |  | Interviewed questionnaire of patient and/or caregiver |
| Additional secondary outcome measures |  |  |  |  |  |  |  |
| Motor symptoms | MDS-UPDRS part III, motor score in on-drug phase | X |  |  | X |  | Physical examination (blinded) |
|  | MDS-UPDRS part II (1), motor experiences of daily living in off-drug phase | X |  |  | X |  | Self-reported questionnaire of patient and caregiver |
| *Table 2 continued* |  |  |  |  |  |  |  |
| **Outcome** | **Assessment^1^** | **Assessments per visit** | | | | | **Data collection method** |
|  |  | *Visit*  *1*  *Baseline* | *Visit 2** | *Visit 3*  *15w* | *Visit 4*  *30w* | *Visit*  *5*  *52w* |  |
|  | MDS-UPDRS part II, motor experiences of daily living in on-drug phase | X |  |  | X |  | Self-reported questionnaire of patient and caregiver |
|  | Clinical Dyskinesia Rating Scale (5) | X |  |  | X |  | Physical examination |
|  | Motor symptom diary | X |  |  | X |  | Proxy-report by caregiver |
| Cognition | Montreal Cognitive Assessment (6) | X |  |  | X |  | Cognitive examination |
| Functional health status | ALDS (7) - in off-drug phase | X |  |  | X |  | Interviewed questionnaire of patient and/or caregiver |
|  | ALDS - in on-drug phase | X |  |  | X | X | Interviewed questionnaire of patient and/or caregiver |
|  | Modified Rankin Scale (8) | X |  |  | X |  | Interview of patient and/or caregiver |
|  | Hoehn and Yahr stage (9) | X |  |  | X |  | Interview of patient and/or caregiver |
|  | Clinical Global Impression of Change | X |  |  | X |  | Interview of patient and/or caregiver |
| Falls | Trial specific questionnaire | X |  | X | X |  | Interview of patient and/or caregiver |
| Parkinson’s Disease and Psychiatric Medication use | Trial specific questionnaire | X |  | X | X |  | Interview of patient and/or caregiver |
| (Serious) adverse events | Trial specific questionnaire |  | X | X | X |  | Interview of patient and/or caregiver |
|  |  |  |  |  |  |  |  |
| *Table 2 continued* |  |  |  |  |  |  |  |
| **Outcome** | **Assessment^1^** | **Assessments per visit** | | | | | **Data collection method** |
|  |  | *Visit*  *1*  *Baseline* | *Visit 2** | *Visit 3*  *15w* | *Visit 4*  *30w* | *Visit*  *5*  *52w* |  |
| Caregiver burden | Zarit Burden Interview (10) | X |  | X | X |  | Interview of caregiver |
| Medical care use | Trial specific questionnaire |  |  |  | X |  | Interview of patient and/or caregiver |
| Other study parameters |  |  |  |  |  |  |  |
| Presence of (long-lasting) delirium | DSM-5^3^ |  | X |  |  |  | Interview of patient and/or caregiver |
| Treatment satisfaction | Trial specific questionnaire |  |  |  | X |  | Self-reported questionnaire of patient and caregiver |
| *Table 2 continued* |  |  |  |  |  |  |  |

*ALDS, Academic Medical Center Linear Disability Score; DSM-5, Diagnostic and Statistical Manual of Mental Disorders, fifth edition; A-IADL-Q-SV, Amsterdam iADL questionnaire – short version; MDS-UPDRS, Movement Disorder Society - Unified Parkinson’s Disease Rating Scale.*

* Visit 2 only applies to patients randomized to the DBS group and will take place one week after the surgery.

1. All assessments are performed in on-drug phase unless otherwise specified.
2. Baseline characteristics include age, sex, medication use, age at onset of PD, time since PD diagnosis, time since dementia diagnosis, and time between diagnosis of PD and first symptoms of dementia.
3. Patients will be diagnosed according to the diagnostic criteria for delirium in DSM-5. Long-lasting delirium is defined a delirium lasting longer than one week.

**References**

1. Goetz CG, Fahn S, Martinez-Martin P, Poewe W, Sampaio C, Stebbins GT, et al. Movement Disorder Society-sponsored revision of the Unified Parkinson’s Disease Rating Scale (MDS-UPDRS): Process, format, and clinimetric testing plan. Mov Disord. 2007 Jan;22(1):41–7.

2. Jutten RJ, Peeters CFW, Leijdesdorff SMJ, Visser PJ, Maier AB, Terwee CB, et al. Detecting functional decline from normal aging to dementia: Development and validation of a short version of the Amsterdam IADL Questionnaire. Alzheimer’s Dement Diagnosis, Assess Dis Monit. 2017;8:26–35.

3. Cummings JL, Mega M, Gray K, Rosenberg-Thompson S, Carusi DA, Gornbein J. The Neuropsychiatric Inventory: Comprehensive assessment of psychopathology in dementia. Neurology. 1994 Dec 1;44(12):2308–2308.

4. Weintraub D, Hoops S, Shea JA, Lyons KE, Pahwa R, Driver-Dunckley ED, et al. Validation of the questionnaire for impulsive-compulsive disorders in Parkinson’s disease. Mov Disord. 2009 Jul 30;24(10):1461–7.

5. Hagell P, Widner H. Clinical rating of dyskinesias in Parkinson’s disease: Use and reliability of a new rating scale. Mov Disord. 1999 May;14(3):448–55.

6. Nasreddine ZS, Phillips NA, BÃ©dirian V, Charbonneau S, Whitehead V, Collin I, et al. The Montreal Cognitive Assessment, MoCA: A Brief Screening Tool For Mild Cognitive Impairment. J Am Geriatr Soc. 2005 Apr;53(4):695–9.

7. Holman R, Lindeboom R, Glas C, Vermeulen M, de Haan R. Constructing an item bank using item response theory: the amc linear disability score project. Heal Serv Outcomes Res Methodol. 2003;4(1):19–33.

8. van Swieten JC, Koudstaal PJ, Visser MC, Schouten HJ, van Gijn J. Interobserver agreement for the assessment of handicap in stroke patients. Stroke. 1988 May;19(5):604–7.

9. Hoehn MM, Yahr MD. Parkinsonism: onset, progression, and mortality. Neurology. 1967 May 1;17(5):427–427.

10. Zarit SH, Reever KE, Bach-Peterson J. Relatives of the Impaired Elderly: Correlates of Feelings of Burden. Gerontologist. 1980 Dec 1;20(6):649–55.
